# Economic Recession Affects Gambling Participation But Not Problematic Gambling: Results from a Population-Based Follow-up Study

**DOI:** 10.3389/fpsyg.2017.01247

**Published:** 2017-07-25

**Authors:** Daniel T. Olason, Tobias Hayer, Gerhard Meyer, Tim Brosowski

**Affiliations:** ^1^Department of Psychology, University of Iceland Reykjavík, Iceland; ^2^Institute of Psychology and Cognition Research, University of Bremen Bremen, Germany

**Keywords:** gambling, problem gambling, prevalence, economic recession, longitudinal study

## Abstract

In October 2008, Iceland experienced the fastest and deepest financial crisis recorded in modern times when all three major banks went bankrupt in less than 2 weeks. The purpose of this follow-up study is to examine potential changes in participation in 12 different gambling types and in problem gambling before (time 1; year 2007) and after (time 2; year 2011) the economic collapse in 2008. The time between the first and second wave of data collection was 3.5 years. In total, 1,531 participants took part in the study, 688 males and 843 females. There was a considerable increase in past year gambling behavior from 2007 to 2011, mostly due to increased participation in lotto (National lotto and Viking lotto) but also in bingo, monthly lotteries (class lotteries with at least monthly draw) and scratch tickets. Only EGMs (electronic gaming machines) participation declined significantly between the two timepoints. Examining past year problematic gambling figures revealed that there were no changes in the prevalence figures between the year 2007 (1.2%) and 2011 (1.1%). Further examination revealed that those who reported financial difficulties due to the recession were more likely to buy lotto- or scratch tickets during the recession than those who were not financially affected by the crisis. These findings remained after controlling for background variables and baseline gambling activity (gambling in 2007). Overall, the findings of the follow-up study suggest that when people are experiencing financial difficulties during economic recessions, the possibility to improve their financial situation by winning large jackpots with low initial stakes becomes more enticing.

## Introduction

The global bank crisis of 2008 resulted in dramatic economic and political changes for the Icelandic population. Following the privatization of the three largest banks in the early 2000s, the economy experienced unprecedented growth rates, largely driven by the activities of the financial sector (Benediktsdottir et al., 2011). The assets of the three major banks grew sevenfold from the year 2003 to 2007, with a great influx of foreign capital pouring into the country and leading to the offering of easy loans to the public (Ragnarsdottir et al., 2013). Standard of living increased during this period and in spite of increasing household debts raised the expectation of the public toward more prosperity in the years to come (Matthiasson, 2008; Ragnarsdottir et al., 2013). However, as this growth was mostly financed with short-term loans available to the banks on the international market, the financial situation of the banks became unsustainable when their availability dried up in the global bank crisis. In less than 2 weeks in October 2008, the three largest banks in Iceland went bankrupt and in the following months the majority of other financial institutions were bankrupted as well (Benediktsdottir et al., 2011; Schwartz, 2011; Bergman, 2014; Johnsen, 2014). In fact, Iceland experienced the fastest and deepest financial crisis recorded in modern times. Within a short period of time, the financial crisis wiped out the stock market, the national currency lost more than half its value, there was a sharp increase in inflation rates resulting in huge losses (for both businesses and the general public) and increases in both household debts (indexed to inflation rates) and in prices for domestic goods (Danielsson and Zoega, 2009; Benediktsdottir et al., 2011; Statistics Iceland, 2011).

The enormity of the Icelandic economic collapse and the following sharp decline in standard of living has challenged researchers to study the effects of a sudden economic crash on population behavior and well-being. For example, a longitudinal study on a national cohort of 3.755 adults who responded to a survey on health and lifestyles in 2007 (prior to the crisis) and again in 2009 showed that women reported increased signs of stress during the period, but the effect was not found for men (Hauksdottir et al., 2013). This finding concurs with an earlier study examining attendance at emergency departments in Reykjavik shortly after the economic meltdown in October 2008 (Guðjónsdóttir et al., 2012). It was found that compared to the same timeframe in 2006 and 2007 there was an increase in the total number of visits to the cardiac emergency department, particularly among women. Furthermore, a study on sickness and sickness absence among 2.356 employees within the educational (teachers and kindergarten teachers) and care services (elderly and disabled care) revealed that sickness and sickness absence increased substantially over a 3-year period (2010–2013), but importantly more so within workplaces who had been subjected to downsizing during the financial crisis (Sigursteinsdóttir and Rafnsdóttir, 2015).

### Gambling and Recessions

Within the gambling literature empirical studies on the effects of economic recessions on gambling patterns are lacking and existing approaches are mainly based on aggregated expenditure or sales data. For example, results from studies in the United States suggest that economic recession results in decreased participation in casino type games (including EGMs-electronic gaming machines) but at the same time in stable or even increased lottery sales (Horváth and Paap, 2012; Lyons, 2013). More recently, two studies from Sweden and Ireland using repeated cross-sectional household data (collected before and after the most recent economic recession) show similar findings (Rude et al., 2014; Eakins, 2016). The study from Sweden, using household data on general gambling expenditure collected in 2003 and again in 2009, found that the decision to gamble was affected by the recession (Rude et al., 2014). The Irish findings showed that bookmaker/toto participation was hampered during the recession, but lottery participation was not (Eakins, 2016). In general, these studies suggest that lotteries are recession-resistant but casino type games (including EGMs) and bookmaker betting are not. However, it has to be kept in mind that the evidence is only based on aggregated sales or expenditure data on relatively few gambling types. Such aggregated data does not offer the opportunity to examine directly if individual financial difficulties are related to changes in gambling behavior during an economic recession.

A repeated cross-sectional gambling study, incorporating data from both before and after the Icelandic economic meltdown in 2008 (*N* = 8.249), examined the effects of the economic crisis on participation in 11 different types of gambling as well as problem gambling (Olason et al., 2015). Firstly, the findings revealed a modest increase in problematic gambling over the study period, but further examination of the data suggested that this was most likely due to an increase in card and Internet gambling among young men. More interestingly, the main findings suggested a general increase in past year gambling within the Icelandic population which extended to most types except betting on the outcome of sport events, skill games and EGMs. In fact, a significant reduction in participation was only found for EGMs gambling. In contrast, the most notable increase in gambling participation was observed for the most popular gambling type, lotto. Further analyses, examining the association between financial difficulties and gambling, revealed that those participants who experienced financial problems due to the economic crisis were 52% more likely to have bought a lottery ticket during the recession period compared to those who were not affected financially by the crisis. Thus, this study extended the findings from the United States (Horváth and Paap, 2012; Lyons, 2013), Sweden (Rude et al., 2014), and Ireland (Eakins, 2016) by not only examining more types of gambling but also by addressing the individual effects of economic hardship on gambling during an economic crisis directly. In general, it also replicated the common finding that lotteries seem recession-resistant but EGMs do not.

### Main Aims of the Present Study

It is notable that all existing studies that have examined the potential effects of economic crisis on gambling behavior so far are based on repeated cross-sectional study designs. Therefore, the estimates for change over time are based on comparisons of data from different samples collected at different time points. Consequently, a cross-sectional study design cannot exclude the possibility that changes over time might be partly or totally explained with inter-individual differences in reaction or experience toward certain events, such as an economic crisis. This problem is avoided using a longitudinal design as it allows for the study of intra-individual changes in gambling behavior between different time points, following the same people. In general, a longitudinal study design provides a more sensitive test of the effects of a macro-environmental event, such as a major economic crisis, compared to a repeated cross-sectional study design (Slutske, 2007).

The economic crisis in Iceland therefore created a unique opportunity to examine the effects of the economic collapse on gambling behavior using two study designs, a repeated cross-sectional as well as a longitudinal one. Thus, in 2011 two studies were conducted, a third cross-sectional study and the present follow-up study on participants who had taken part in a gambling study from 2007. This gave us the possibility to compare findings on changes in gambling behavior from 2007 to 2011 between two different research designs (see Olason et al., 2015, for the results of the repeated cross-sectional study). Using follow-up data, the main aim of this study is to examine the potential changes in gambling participation and problematic gambling among the Icelandic population before (2007) and after (2011) the economic crisis in 2008. The same methodology and measures for gambling and problem gambling were applied at both time points. *A priori*, we expected to replicate the previous findings of Olason et al. (2015) with a general increase in total gambling figures that will extend to most gambling types, except EGMs. Secondly, Olason et al. (2015) reported that those who experienced financial difficulties due to the economical collapse were more likely to buy lotto tickets during the economical crisis than those who were not financially affected by the crisis, and this effect held after adjusting for gender, age, and education. The longitudinal design of the present study offers a more stringent test of this finding by adjusting not only for gender, age and education but also for baseline levels (2007) of gambling activities. Finally, the study design also offers, for the first time, the chance to evaluate problem gambling trajectories in Iceland.

## Materials and Methods

### Participants and Procedure

In 2007, a total of 3.004 18- to 70-year-old Icelanders drawn randomly from the National Registry participated in a national gambling study. Information was collected via telephone interviews conducted by trained interviewers at the Social Science Institute at the University of Iceland. Subsequently, respondents were invited to participate in a follow-up study at a later time. In total, 1.780 participants (59.2%) agreed to participate in the follow-up study. The data for the second wave was again collected by telephone by the Social Science Institute in 2011. In total, information was obtained from 1.531 participants or 86% of those who agreed to participate in the follow-up study. The time between the first and second wave of data collection was 3.5 years.

The study was carried out in accordance with the 1964 Helsinki Declaration and its later amendments. The study was reported to the Data Protection Authority of Iceland and in accordance with their recommendation we collected written informed consent from all participants in the follow-up study. Further, all participants were assured of confidentiality and anonymity and informed that they could withdraw their participation at any time or choose to not answer individual questions.

### Instruments

The structured questionnaire included a total of 180 questions (not all relevant to the present study) that were presented to the participants as follows: First, participants were asked about their gambling behavior. If a participant had gambled at least once during the past 12 months in any gambling activity he was also presented with questions on problem gambling and motivations to gamble. Subsequently, a number of questions regarding gambling related-attitudes and his background (e.g., age, education, marital status, work, and income) were presented. At the end of the follow-up interview, the participants were asked questions regarding the effects of the economical collapse on their finances and well-being. The number of questions for each participant could vary depending on their gambling activities. On average each participant answered 55 questions.

(1) Gambling behavior: At both data collection points, gambling behavior was measured with the same methodology. First, respondents were asked if they had participated in a gambling type in the past 12 months and if they responded positively they were asked how frequently they had gambled in that gambling type on a scale ranging from 1 (few times in the last 12 months), 2 (one to three times a month), 3 (one to two times a week), 4 (three to six times a week), and 5 (daily). Participants were asked at both data collection points about their participation in licensed gambling activities such as lotto (National lotto and Viking lotto), scratch tickets, EGMs (electronic gaming machines), sport pools, sport betting (fixed odds sport betting on individual games), live sport betting (live betting on in-play events of sport events, e.g., the first corner in football matches), monthly lotteries (three class lotteries with monthly draws) and bingo: Also, participants were asked about their participation in non-licensed offline gambling activities such as poker (with cards), illegal casinos and betting on games of skill (e.g., billiard, golf, bowling). Finally, all participants were asked about their participation in non-licensed gambling activities on online international Internet gambling sites (e.g., scratch tickets, EGMs, sport betting (including both fixed odds sport betting and live sport betting), Internet poker and other casino gambling activities).
(2) Problem gambling: The Problem Gambling Severity Index (PGSI; Ferris and Wynne, 2001) is a nine-item instrument specifically devised to measure problem gambling in general populations. The time frame refers to the past 12 months and for each item, respondents answer on a four-point scale (0 = never, 1 = sometimes, 2 = most of the time and 3 = almost always). All respondents who gambled at least once during the past 12 months answered the PGSI. The total score ranges from 0 to 27 where a score from 8 or more signifies problem gambling, a score from 3 to 7, moderate risk gambling, a score from 1 to 2 low risk gambling and a score of 0 non-problem gambling. To ensure comparability with the repeated cross-sectional study (Olason et al., 2015) we combined the moderate risk gambling and problem gambling groups into one group signifying problematic gambling (score 3+ on PGSI). Psychometric evaluations of the instrument in different cultures suggest that the PGSI is a reliable and valid measure of problem gambling (Ferris and Wynne, 2001; Holtgraves, 2009; Loo et al., 2011; Sharp et al., 2012). Two independent Icelandic translations of the PGSI were initially generated, the versions were then examined and one final version obtained. A professional translator retranslated the final version which was then compared to the original English version to ensure accuracy. The factor structure and reliability of the PGSI were examined in a sample of 1,266 university students and the results revealed a uni-dimensional scale with acceptable reliability (α = 0.84) (Olason et al., 2003).
(3) Economic collapse: An effort was made to evaluate the influence of the economic collapse on participants’ finances. However, due to the overall length of the questionnaire (180 questions), a detailed examination of this subject was not possible. Therefore, a decision was made to use one question on financial well-being obtained from Statistic Iceland that has been used in other national surveys on health and wellbeing of Icelanders during the economic collapse in Iceland as well (Statistics Iceland, 2011; Gudmundsdottir, 2013). Participants were asked about their financial difficulties in relation to the economic collapse during the last 12 months answering on a five-point scale ranging from 1 (very easy) to 5 (very difficult): “How easy or difficult has it been for you and/or your family to make ends meet financially every month, for example paying for accommodation, food and other monthly bills?”

### Statistical Analysis

Initial analyses revealed that Internet participation on non-licensed Internet gambling sites was rather infrequent for different types of gambling: Therefore all questions on non-licensed Internet gambling were combined into one variable signifying total Internet gambling for both data collection points.

Initially, at baseline (year 2007) four age groups (18–25, 26–40, 41–55, and 56–70 years) were generated according to the convention in Icelandic gambling research reflecting different life cycles. However, due to the longitudinal nature of the present study, we added 3 years to each age brackets to reflect the increased age of participants at the follow-up (year 2011). Thus, the age groups were the following; 21–28, 29–43, 44–58, and 59–74 years.

A preliminary examination of the distribution of answers on the proxy question for the effects of the financial collapse indicated that 48.5% of participants found it easy (1 = very easy; 2 = rather easy) to make ends meet, 19.6% neither easy nor difficult (3 = neither/nor) and 31.5% found it difficult (4 = rather difficult; 5 = very difficult). To ensure comparability with the earlier repeated cross sectional study (Olason et al., 2015) and to differentiate clearly between the groups who either experienced financial difficulties or not, the middle category (those who found it neither easy or difficult) was excluded.

In order to check the association of the proxy variable (financial difficulties) with an increased probability of participation in certain types of gambling in 2011, successive logistic regression models were built up. In a first step, for each type of gambling, simple logistic regression models (model 1 – simple regression) were executed to predict the probability of participation in 2011 by the proxy in 2011. If a statistically significant association between the proxy and the gambling type existed, we also examined alternative explanations by complementing the simple predictive model with effects of gender, age and education (model 2 – adjusted for demographics) as well as by adding effects of past participation in the given type of gambling in 2007 (model 3 – adjusted for demographics and past behavior). If the proxy showed a statistically significant association with a type of gambling even after adjusting for demographic variables and past gambling behavior, there hardly remained any alternative explanation for the increased probability of participation in 2011 than the proxy itself. Changes in metric variables between 2007 and 2011 were analyzed with repeated analyses of variance, adjusted for inter-individual effects of gender, age, and education.

## Results

### Changes in Gambling from 2007 to 2011

Trends in total gambling are shown in **Table 1**. The results reveal that gambling participation had increased, as an additional 10.7% of the sample reported gambling in 2011 compared to 2007. Further, there was also a significant increase in the number of gambling types played, rising from 1.3 games in 2007 to 1.6 games in 2011 [*t*(1,530) = 10.08, *p* < 0.001]. Trends for total gambling were similar for gender and educational levels but more variation was observed for different age groups (see **Table 1**).

**Table 1:** Total gambling trends over time by demographics.

	2007 %	2011 %	McNemar Chi-square	% Change
**All participants (*n* = 1,531)**	68.8	79.5	84.01^∗∗∗^	+10.7
				
**Gender**				
Males (*n* = 689)	69.8	79.4	31.53^∗∗∗^	+9.6
Females (*n* = 842)	67.9	79.6	51.69^∗∗∗^	+11.7
**Age groups**				
21–28 (*n* = 156)	59.0	67.9	3.52	+8.9
29–43 (*n* = 468)	68.6	83.8	45.79^∗∗∗^	+15.2
44–58 *n* = 565)	68.8	78.8	27.01^∗∗∗^	+10.0
59–74 (*n* = 342)	73.4	80.1	9.87^∗∗^	+6.7
**Education**				
Primary (*n* = 274)	74.1	84.3	15.19^∗∗∗^	+10.2
Secondary (*n* = 589)	71.5	82.3	34.21^∗∗∗^	+10.8
University (*n* = 655)	64.1	75.4	36.01^∗∗^	+11.3

*^∗∗^*p* ≤ 0.01, ^∗∗∗^*p* ≤ 0.001.*

In order to identify possible interaction effects between demographic variables and total gambling the data were entered into repeated measures mixed ANOVAs using a four point (0 = never gambled to 3 = gambled weekly) measurement for total gambling frequency as the dependent variable at time 1 and 2 and demographic variables as between group variables. The results revealed that the interaction was neither significant between time and gender [*F*(1,1,529) = 0.684, *p* > 0.05], nor between age groups [*F*(3,1,527) = 0.480, *p* > 0.05] or educational levels [*F*(2,1,515) = 0.617, *p* > 0.05]. However, the overall effects for between group analyses revealed significant differences for age groups [*F*(3,1,527) = 11.060, *p* < 0.001] and educational levels [*F*(2,1,515) = 10.096, *p* < 0.001] but not for gender [*F*(1,1,529) = 1.507, *p* > 0.05]. As can be seen in **Table 1**, at both time points the youngest age group was less likely to gamble than older age groups (2007: [χ(3,1,531) = 10.38, *p* < 0.05]; 2011: [χ(3,1,531) = 18.24, *p* < 0.001]). Further, those with university education were at both time points less likely to gamble than those with lower levels of education (2007: [χ(2,1,518) = 12.20, *p* < 0.01]; 2011: [χ(2,1,518) = 13.55, *p* < 0.01]).

We then explored in detail to what extent changes in gambling could be observed for different types of gambling (see **Table 2**). The greatest changes were found for lotto where an additional 16% of the sample reported buying lotto tickets in 2011 compared to 2007. Also, participating in bingo, monthly lotteries and buying scratch tickets was more common in 2011 than in 2007. Interestingly, a significant decrease was observed for EGMs.

**Table 2:** Gambling trends over time for different gambling types.

	2007 %	2011 %	McNemar Chi-square	% Change
Lotto	49.7	65.7	154.64ˆ***	+16.0
Bingo	4.8	10.8	42.86ˆ***	+6.0
Monthly lottery	33.8	37.2	12.37ˆ***	+3.4
Scratch tickets	16.3	19.6	8.12ˆ**	+3.3
EGMs	7.3	4.8	13.93ˆ***	–2.5
Poker	5.1	5.6	0.39	+0.6
Football pools	6.3	6.9	0.86	+0.6
Sport betting	3.6	3.1	0.73	–0.5
Games of skill	1.6	1.6	0.0	0.0
Live sport betting	0.5	0.2	0.34ˆ#	–0.3
Illegal casinos^1^	0.3	0.3	1.00ˆ#	0.0
Internet gambling^2^	1.1	1.1	1.00ˆ#	0.0

*^1^Small underground establishments where you play mostly cash poker, black jack, or roulette. ^2^Gambling on non-licensed international websites. # Binominal distribution used. ^∗∗^*p* ≤ 0.01, ^∗∗∗^*p* ≤ 0.001.*

To examine potential interaction effects between demographic variables and changes in gambling frequency in different gambling types the data were again entered into a series of repeated measures mixed ANOVAs using a four-point (0 = never gambled to 3 = gambled weekly) measurement for each gambling type as the dependent variable at both time points and demographic as between group variables. Live sport betting, illegal casinos, and Internet gambling were excluded from these analyses as too few individuals participated in these activities. Prior to these analyses the significance levels were adjusted for the high number of tested interaction effects (*n* = 27) using the Bonferroni correction, resulting in significance levels of *p* ≤ 0.001.

The results revealed that the interactions between time and gender, time and educational levels and time and age groups were not significant for any of the different gambling types (*p* > 0.001).

### Changes in Problematic Gambling

In 2007, 4.8% of all participants were low risk gamblers and 1.2% problematic gamblers (score 3+ on the PGSI). Figures were slightly lower in 2011 with 4.2% low risk gamblers and 1.1% problematic gamblers. To check for changes in levels of problem gambling we first calculated if the total scores on PGSI had changed from 2007 to 2011 and subsequently tested for interaction effects for background variables using repeated measures mixed ANOVAs. No overall change between PGSI scores from 2007 to 2011 was found [*t*(1,529) = 1.378, *p* > 0.05] and interaction effects with gender [*F*(1,1,528) = 3.33, *p* > 0.05], age groups [*F*(3,1,526) = 0.477, *p* > 0.05] and educational levels [*F*(2,1,514) = 1.62, *p* > 0.05] were all non-significant.

Subsequently, we examined the consistency of individual trajectories from 2007 to 2011 (see **Table 3**). Interestingly, there was a considerable shift over time for different gambling groups. Specifically, about half of those who did not gamble in 2007 did so in 2011 and 0.4% (*n* = 2) were found to be problematic gamblers. In fact, over half (*n* = 9) of those who had gambling-related problems in 2011 did not have any problems in 2007 (see column 5 in **Table 3**).

**Table 3:** Consistency of individual trajectories of gambling groups.

	Gambling groups in 2011			
Gambling		Non-problem	Low risk	Problematic
groups	Non-gamblers	gambling	gambling	gambling
in 2007	*n* (%)	*n* (%)	*n* (%)	*n* (%)
Non-gamblers	**238 (49.8)**	232 (48.5)	6 (1.3)	2 (0.4)
Non-problem gamblers	68 (7.1)	**851 (88.6)**	34 (3.5)	7 (0.7)
Low risk gamblers	7 (9.5)	42 (56.8)	**22 (29.7)**	3 (4.1)
Problematic gamblers	1 (5.6)	9 (50)	3 (16.7)	**5 (27.8)**

*Consistency rates are presented in boldface.*

Consistency rates (figures are bolded in **Table 3**) were high for non-problematic gamblers with 88.6%, but little consistency was observed for the low risk and problematic gambling groups. Less than a third of those who had problems in 2007 were still categorized as such in 2011. Further, over 50% of those who were defined as problematic gamblers in 2007 had either quit gambling or gambled without any problems in 2011. Further examination of the data revealed that only one participant had received treatment for his gambling problems in 2007 and three reported having done so in 2011. Of those three individuals, two were categorized as non-problem gamblers and one was still a problematic gambler.

### Effects of the Economic Crisis on Gambling and Problem Gambling

Initial analysis with regard to the relationship between financial difficulties and demographic variables revealed that females (44.6%) were more likely than males (32.6%) to report financial difficulties [χ(1,1,215) = 18.31, *p* < 0.001], and those with primary education (53.0%) were more likely to report financial difficulties than those with secondary (38.7%) or university (34.3%) education [χ(2,1,208) = 22.61, *p* < 0.001].

The results of the simple logistic regression analysis using financial difficulties as a predictor revealed that that those who experienced financial difficulties were more likely to have gambled at least once in 2011 (OR = 1.68, *p* < 0.01), to have bought lotto tickets (OR = 1.77, *p* < 0.01) and scratch tickets (OR = 1.55, *p* < 0.01) than those who did not experience any financial difficulties during the 12 months before the study (see model 1 in **Table 4**).

**Table 4:** Odd ratios for financial difficulties.

Dependent variable	Model 1: Simple regressions	Model 2: Adjusted for demographics	Model 3: Adjusted for demographics and past gambling behavior
Total gambling	1.68^∗∗^ (1.25; 2.27)	1.64^∗∗^ (1.21; 2.23)	1.60^∗∗^ (1.13; 2.27)
Lotto	1.77^∗∗^ (1.38; 2.28)	1.76^∗∗^ (1.36; 2.28)	1.46^∗^ (1.08; 1.98)
Scratch tickets	1.55^∗∗^ (1.17; 2.06)	1.53^∗∗^ (1.14; 2.04)	1.41^∗^ (1.03; 1.94)
Poker	0.78ns (0.46–1.33)		
Football pools	0.94 ns (0.59; 1.49)		
Sport betting	1.01ns (0.52; 1.96)		
Monthly lotteries	1.01ns (0.79; 1.28)		
EGMs	1.04ns (0.61; 1.76)		
Games of skill	0.72ns (0.29; 1.78)		
Bingo	0.96ns (0.66; 1.39)		
Internet gambling	0.89ns (0.26; 3.04)		
Illegal casinos	3.11ns (0.28; 34.4)		
Live sport betting	0.00ns (0.00; 0.00)		
Problematic gambling	1.56ns (0.54–4.48)		

*ns, non-significant. ^∗^*p* ≤ 0.05, ^∗∗^*p* ≤ 0.01.*

The results for the more restricted models revealed that even after controlling for gender, age, education, and past gambling behavior (model 3 in **Table 4**) the effects remained significant. All three final models showed a statistically significant increase of the log-likelihood-ratio (*p* ≤ 0.01).

Finally, we tested if those who started gambling between the two time points (i.e., those who gambled in 2011 but not in 2007) were different from those who did not gamble at all (at neither time point) in terms of financial difficulties. The results showed that those who started to gamble between 2007 and 2011 were more likely to have difficulties to make ends meet (41.6%) than those who did not gamble (28.4%) at all [χ(1,380) = 7.23, *p* < 0.01].

## Discussion

The sudden collapse of the three major banks in the beginning of October 2008 in Iceland started a major financial crisis in the country that dominated the public discussion in the media. The first 3 years of the crisis, from 2008 to 2011, thus consisted of substantial changes in the society, including considerable decreases in the living standards of the population. This situation not only led to increased distress, sickness, and sickness absence among employees subjected to downsizing (Snorradóttir et al., 2013; Sigursteinsdóttir and Rafnsdóttir, 2015), but also to changes in dietary habits, stress levels, and travel behavior of the population (McClure et al., 2013; Ásgeirsdóttir et al., 2014; Ulfarsson et al., 2015).

The economic crisis in Iceland therefore created a unique opportunity to also look at the effects of the economic collapse on gambling behavior using two study designs, a repeated cross-sectional study (Olason et al., 2015) and the present longitudinal study.

### Gambling Behavior

As expected, the findings of the follow-up study in general confirmed the findings of the repeated cross-sectional study (Olason et al., 2015) showing a considerable increase in total gambling figures (about 11 percentage points), mostly due to increased participation in lotto (16 percentage points), but also in other types of lotteries such as bingo, monthly lotteries, and scratch tickets. Further, there were no changes in betting on the outcomes of sport events (sport pools, sport betting), poker or Internet gambling but a significant decrease in EGMs participation.

It is noteworthy that increases in gambling during economic recession was only found for lotteries (e.g., lotto, scratch cards, monthly lottery, and bingo) but not in other types of gambling. Further, examining potential gender differences in total gambling and in different gambling types over time revealed no significant differences for men and women. This lack of significant interaction effects can be explained by the fact that meaningful changes over time were only found for gambling types, where past studies have shown small or no differences between male and female participation (Olason, 2008, 2012; Olason et al., 2015). Participation in gambling types such as EGMs, sport gambling, Internet gambling, or poker, typically preferred by males, did, however, not change during the recession. Thus a lack of gender differences in changes of gambling participation over time is probably based on the fact that both genders equally increased their participation in gambling types that typically show little gender differences overall.

Considering individuals’ decisions to gamble, it is likely that a number of factors influences this decision, including the choice of participating in particular gambling activities. Thus motivations for gambling may differ and can vary depending on individual preferences. Common motivations for gambling reported in the literature are related to winning money, entertainment, enjoyment, excitement, escape, social interaction, and supporting charities (Ariyabuddhiphongs, 2011; Binde, 2013). Other factors that also might be important are the time invested in the gambling activity, knowledge required to play the game and the perceived risks associated with a certain gambling type (Eakins, 2016). To gamble on sport events and poker requires both time and knowledge and earlier Icelandic studies have shown that people perceive EGMs, poker and sports gambling as more addictive than lotteries (Olason, 2008, 2012). Taken together, lotteries not only take a relatively short time to play and require little knowledge, but are also perceived of as low-risk gambling activities. In fact, lotteries are ubiquitous and thus individuals tend not to regard them as gambling, but rather as a form of a leisure activity (Ariyabuddhiphongs, 2011). Further, the added benefits of lotteries encompass that playing requires only a small initial stake and a possibility (although very small) of winning large jackpots. It is therefore perceivable that lotteries are regarded as a more acceptable type of gambling during recessions, when money is scarce. This is in fact supported by our findings: Participants who experienced financial difficulties during the recession were overall more likely to have bought lotto- or scratch tickets than those who did not experience financial difficulties. These results remained after controlling for demographics (gender, age, and education) and, more importantly, for past gambling activity. These findings therefore suggest that when people are experiencing financial difficulties during an economic recession they perceive the possibility to win large jackpots with low initial stakes as means to improve their financial situation. This line of argumentation is also supported by the finding that those who did not gamble in 2007 but did so in 2011 (new gamblers) were more likely to report financial difficulties than those who did not gamble at all.

The reported association between financial difficulties and lottery gambling found in the present study is further supported by empirical results of other studies that have also reported significant links between lottery play and income levels (Ariyabuddhiphongs, 2011; Barnes et al., 2011). For example, Welte et al. (2002) found in a household survey in the United States that the bottom three quintiles in socioeconomic status spent the most on the lottery and highest socioeconomic groups spent the least. Earlier, Kearny (2005) reported that with the introduction of state lotteries in the United States households expenditure on non-gambling items (e.g., food, clothes) declined and the response was most pronounced for low-income households. In additions, similar associations with income have been reported in the United Kingdom (Grun and McKeigue, 2000) and Thailand (Ariyabuddhiphongs, 2006).

In general, the changes in gambling behavior found in this study confirm the trends obtained from the repeated cross-sectional study and extend previous findings using sales and expenditure data (Horváth and Paap, 2012; Lyons, 2013; Rude et al., 2014; Olason et al., 2015; Eakins, 2016). Collectively, the results of these studies lead to the conclusion that during an economic recession people tend to continue or even increase their participation in lotteries but decrease their participation in casino type games, including EGMs.

### Problem Gambling

No changes were observed for problematic gambling over time as figures remained just over 1% at both time points. A detailed examination, testing for interaction effects between time and different background variables also revealed no differences in problematic gambling over time for gender, age, or education. These findings are different from those obtained in the repeated cross-sectional study where problematic gambling rose from 1.6% in 2005/2007 to 2.5% in 2011. However, it is important to note that the increase in problematic gambling in that study was prominently found among 18- to 25-year-olds (see p. 765 in Olason et al., 2015). Thus, one probable reason for the differences between the studies might be linked to a maturing out effect for problem gambling. Empirical support for this idea can be found in longitudinal studies on youth to young adulthood (Slutske et al., 2003) or in a recent study from Sweden that showed a higher incidence rate for the age group 16 to 24 compared to 25- to 44-year-olds (Fröberg et al., 2015).

### Stability of Problem Gambling Groups

Prior studies on the stability of problem gambling (tendency to stay at the same diagnostic level over time or intra-individual stability) in non-treatment samples suggest that instead of stability, problem gambling is a fluid condition with multidirectional pathways. That is, movement between problem gambling categories over time are rather common among all age groups, but more often in direction of improvement than worsening of symptoms (e.g., Slutske et al., 2003; Abbott et al., 2004; LaPlante et al., 2008; Delfabbro, 2013; Edgerton et al., 2015; Fröberg et al., 2015).

Although this was not one of the main aims of the present study, the prospective nature allowed us to examine the stability of problem gambling categories in a population-based sample over a 3.5-year period. The results indicate that, as expected, consistency was high for non-problem gamblers with close to 90% of non-problem gamblers in 2007 classified as non-problem gamblers again in 2011. However, the empirical evidence for low risk- and problematic gamblers mirrors the relatively common finding of inconsistency in problem gambling classification over time as less than 30% of the gamblers classified in these groups in 2007 were classified in the same groups in 2011. The trend also suggested improvement rather than worsening in symptoms as the majority of low risk gamblers in 2007 had transferred to the non-problem gambling group (56.8%) or had stopped gambling (9.5%) 3.5 years later. Similarly, for the 2007 problematic gamblers more than half of the group were either gambling without problems (50%) or had quit gambling altogether (5.6%) in 2011. However, the overall problem gambling prevalence did not change over the 3.5-year time period due to the fact that some individuals showed a progression in symptoms from 2007 to 2011 toward problematic gambling (from the non-gambling group: *n* = 2, from the non-problem gambling group: *n* = 7 and from the low risk gambling group: *n* = 3). Taken together, these results, although only based on small case numbers, support prior findings suggesting that problematic gambling in non-treatment samples reflects a pattern of transition and, for the more severe cases, the general trend reflects a decrease instead of stability or an increase in gambling-related problems over time (e.g., LaPlante et al., 2008; Delfabbro, 2013; Edgerton et al., 2015).

There are some limitations of this study worth noting. Firstly, the data is based on self-reports that have the well-known potential limitations related to recall bias among respondents and the possibility that individuals are in general inclined to give deceptive or social desirable answers due to the sensitivity of survey questions. A second limitation refers to the fact that although we received a high retention rate from those who agreed to take part in the follow-up study (86%) the overall response rate from the total sample in 2007 was 51%. However, response rates in surveys among the general public are declining and retention rates in follow-up studies are dropping (e.g., Galea and Tracy, 2007; Arfken and Balon, 2011; Greenberg and Weiner, 2014). The same problem seems to apply to gambling studies (e.g., Williams et al., 2012; Romild et al., 2014; Billi et al., 2015; Meyer et al., 2015). For example, a recent meta-analytic study including 202 published and unpublished international gambling prevalence surveys from 1975 to 2012 found a significant negative association between survey year and response rates. Further, they reported an average response rate for studies using telephone interviews of only 52.5% (Williams et al., 2012). Although the overall response rate of the present study might seem low, it certainly does not differ from the average response rates of other gambling studies (see Williams et al., 2012) and more importantly its effect seems miniscule on gambling participation rates as the overall findings for gambling in the present study concur with the findings from the repeated cross-sectional study conducted over the same time period (Olason et al., 2015). However, the attrition rate are a matter of concern for the current study and other gambling studies with longitudinal design (e.g., Currie et al., 2011; Fröberg et al., 2015).

## Conclusion

The present results show a considerable increase in gambling behavior over a 3.5-year period in Iceland, during one of the most deepest economic crisis in modern times. Certainly, a possible alternative explanation for this trend might be if there were some significant changes in the Icelandic gambling market during the time period of the study, such as new gambling products or increased gambling opportunities. However, the legal gambling market in Iceland remained stable throughout the period, that is no new gambling products were introduced and the availability of existing gambling products did not increase in any way (e.g., Olason and Gretarsson, 2009; Olason, 2012). Our conclusion therefore is that the finding that there is a strong temporal association between individual financial difficulties during such crisis and increased participation in lotto and scratch tickets and the fact that a higher percentage of new gamblers (started gambling during the economic crisis) report financial difficulties than non-gamblers bolster the claim that economic cycles (as a macrosocial factor) and individual gambling behavior are strongly related. Finally, the findings of this follow-up study also extend earlier evidence from repeated cross-sectional studies from Iceland, the United States, Sweden, and Ireland that collectively illustrate that lotteries are recession-resistant but casino gambling (including EGMs) and parimutuel betting are not. In future, longitudinal studies with a broader time frame between measurements need to be conducted to clarify the stability of this relationship.

## Author Contributions

All authors were actively involved with this research paper and all contributed to the writing of the paper. DO is responsible for the data collection, data analysis, and writing of the paper. TH was involved in the overall planning of data analysis and writing of the paper. GM was involved with the writing of the paper and overall planning of the data analysis. TB did statistical analysis and contributed to the writing of the paper.

## Conflict of Interest Statement

The authors declare that the research was conducted in the absence of any commercial or financial relationships that could be construed as a potential conflict of interest.
